# Exercise or sport activities for patients with cancer: A protocol for overview of systematic reviews and meta-analyses

**DOI:** 10.1097/MD.0000000000020084

**Published:** 2020-05-15

**Authors:** Fang-fang Wang, Yang Yuan, Yu-jun Song, Yan-qiong Wu, Yun He, Xiu-xiu Deng, Shui-lin Wu, Ding-mei Dai, Min Wang

**Affiliations:** a School of Basic Medical Sciences, Lanzhou University, Lanzhou; b Hospital of Chengdu University of Traditional Chinese Medicine, Chengdu, Sichuan; c School of Public Health, Lanzhou University, Lanzhou, China.

**Keywords:** cancer, exercise, meta-analyses, overview, sport activities, systematic reviews

## Abstract

**Objective:**

We plan to review all published systematic reviews (SRs) and meta-analyses (MAs) of exercise or sport activities for patients with cancer. The aim of this study is to combine and reanalyze related data and to provide more comprehensive and higher-level evidence.

**Methods:**

We plan to search four English databases and four Chinese databases from inception to June 2019. Patients who were treated by all of exercise or sport activities such as running, gymnastics, taichi, and qigong, will be included. The following information will be extracted from each included SR: first author, year of publication, country of origin, number of primary study; the number of patients enrolled, participant characteristics, duration of cancer diagnosis, cancer types. Preferred Reporting Items for Systematic Reviews and Meta-analyses (PRISMA) and A Measurements Tool to Assess Systematic Reviews 2 (AMSTAR-2) will be used to assess the reporting and methodological quality of SRs/MAs. The characteristics of included SRs/MAs and their quality will be descriptively summarized using systematically structured tables. The network MA approach and narrative synthesis will be used to examine data when applicable. Odds ratio and (standardized) mean difference with their 95% confidence intervals will be used as summary statistics. Stata 13.0 software will be used to analyze and pool data.

**Results:**

The results of the overview will be submitted to a peer-reviewed journal for publication.

**Ethics and dissemination:**

The study is not a clinical study, and we will search and evaluate existing sources of literature. So, ethical approval is not required.

## 1 Introduction

Cancer is the largest risk factor that seriously affects the health of residents. In the recent 10 years, the global burden of cancer has continued to increase. Cancer mortality ranks first among all deaths, which accounting for 25% of all deaths.^[1]^ With the increase in the number of cancer diagnoses and the accompanying decline in mortality in most types of cancer, many patients still live with the physical and psychosocial problems associated with the disease and its treatment, which can endanger their survival and quality of life (QoL).^[2,3,4]^ Some studies have showed that exercise or sport activities can be recommended as part of standard treatment for cancer patients to help prevent and manage physical and psychosocial problems and improve QoL.^[5,6]^

Some previous meta-analyses (MAs) of randomized controlled trials (RCTs) had reported benefits of exercise or sport activities in the cancers treatment.^[7,8,9,10]^ their benefits include improving physical fitness, function, and QoL, reducing fatigue and depression. However, some of RCTs showed that demographic, clinical, and personal factors, such as age, marital status, disease stage, and type of treatment, can influence the effects of sport for patients with cancer. And exercise or sport activities may have different effects on different type of cancers.^[11,12,13]^ Therefore, it is necessary to reanalyze the published systematic reviews (SRs) or MAs.

An overview of SRs/MAs is an increasingly popular method to synthesize evidence and a method of synthesizing a large amount of literature in a particular field,^[14,15]^ and recent years, resources related to overview methods have increased^[16,17,18,19]^ moreover, when SRs/MAs conclusions on an issue are inconsistent, overviews are still the best way to summarize evidence. And overview by assessing the reporting and methodological quality of SRs/MAs to judge the reliability of SRs/MAs evidence, and it could provide higher-level integration of data.

In our overviews, we plan to review all published SRs/MAs of exercise or sport activities for patients with cancer; combine and reanalyze related data; and to provide more comprehensive and higher-level evidence for relevant personnels.

## 2 Methods

### 2.1 Eligibility criteria

This overview has been registered on the International Prospective Register of Systematic Reviews (PROSPERO),^[20]^ with the registration number CRD42019138787. We promise that if protocol amendments occur, the dates, changes, and rationales will be tracked in PROSPERO. In addition, the content of this protocol follows the Preferred Reporting Items for Systematic Review and Meta-analysis Protocols (PRISMA-P) recommendations.^[21]^

#### 2.1.1 Types of reviews

SRs and MAs of data from RCTs, non-RCTs, qualitative studies, and observational studies (such as cohort studies, and case-control studies) that assessed effects of exercise or sport activities for cancers. We will exclude narrative reviews including studies of exercise or sport interventions and other unavailable publications.

#### 2.1.2 Types of participants

Participants will be adult patients with cancers, which are no restrictions to type of cancer, sex, and race. Patients of anyone will be eligible, as long as they received exercise or sport interventions. Patients with hematologic tumor will be excluded.

#### 2.1.3 Types of interventions

We will include all type of exercise or sport activities such as running, gymnastics, taichi, and qigong, among others. These interventions can either be compared to control interventions (standard or usual care) or an exercise or sport activity.

#### 2.1.4 Types of outcome

Primary outcomes are all related to health-related QoL at post-treatment. Among the studies, overall QoL should be measured by scales or tools, such as the Functional Assessment of Cancer Therapy-Breast or Functional Assessment of Cancer Therapy-General (FACT-B or FACT-G), Karnofsky Performance Status (KPS) score, European Organization for Research and Treatment of Cancer Quality of Life Questionnaire-Core 30, and the Medical Outcomes Study Short Form-36 (SF-36), etc. Secondary outcomes include cancer-related symptoms and therapy-related adverse events such as pain, flushes, fatigue, sleep disturbance, hair loss, negative mood, diarrhea, flatulence, nausea, and vomiting.

### 2.2 Search strategy

We plan to search the following four English databases and four Chinese databases from inception to June 2019: PubMed, Embase, Cochrane Library, Web of Science, WanFang database, China Academic Journals Full-text database, China Biomedical Literature database, and VIP database. The search strategy for each database used a combination of subject headings and free-text keywords to describe the exercise or sport activities, cancer, and SRs/MAs. The language restricted as English and Chinese. The manual searches of reference lists will be carried out to expand the included studies. The search strategy of PubMed as an example is shown in Table 1. The search terms will be adapted for use with other bibliographic databases in combination with database-specific filters, where these are available.

**Table 1:**
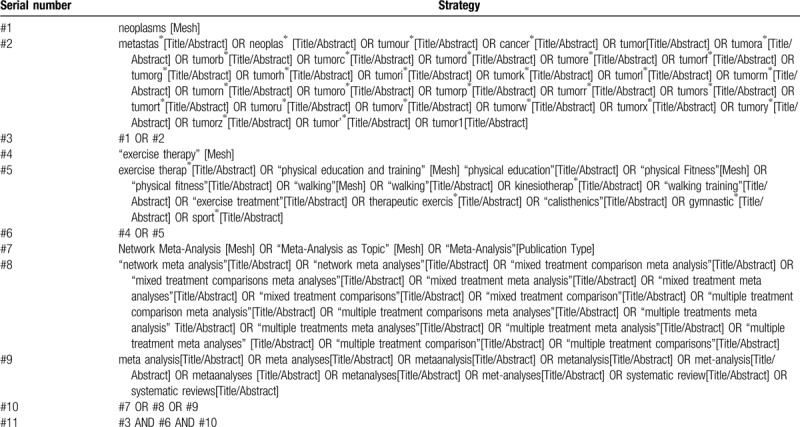
The search strategy in PubMed.

### 2.3 Selection of reviews

**Figure 1:**
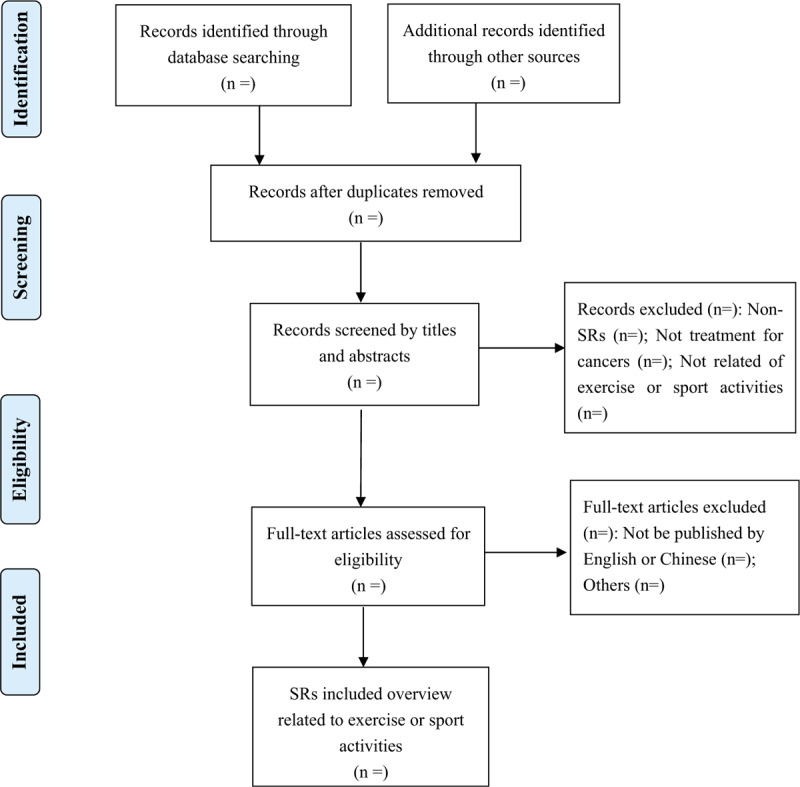
Selecting flowchart of systematic reviews related to exercise or sport activities.

Following initial removal of duplicate and nonrelevant records, 2 authors will independently screen search results (based on abstract and title) against inclusion criteria for full-text review. Full text of publications will be further screened for eligibility. A bespoke assessment of study eligibility form will be used for documentation. In the absence of consensus, arbitration by a third author will be sought. References will be managed in Endnote X8.0 (Thomson Reuters). A flow diagram will be used to describe the process (Fig. 1).

### 2.4 Data extraction

Two reviewers will extract data independently using a predesigned data extraction form. Disagreements will be resolved by discussion with a third reviewer. The following information will be extract from each embedded study: first author, year of publication, country of origin, number of primary study, the number of patients enrolled, participant characteristics, duration of cancer diagnosis, cancer types, detailed description of interventions, cancer-related symptoms, QoL, and therapy-related adverse events. If key information could not be obtained from the published reports, then we will contact the review authors or authors of the original reports to provide clarification and further details.

### 2.5 Data synthesis

The characteristics of the included studies and their quality will be descriptively summarized using systematically structured tables established by Excel 2010. We will reexamine data synthesis using a network meta-analysis (NMA) approach and narrative synthesis. First, individual study effect sizes will be synthesized to generate an overall effect size using a random- or fixed-effects model according to the heterogeneity level, weighted by the inverse of variance, and then the NMA will be conducted. For binary outcomes, odds ratio and 95% confidence interval will be used to presented data. For continuous variables, mean difference will be calculated when outcomes measured using the same scale, and the standardized mean difference will be used when different scales were used in different trials, with corresponding 95% confidence interval. Second, if NMAs are not possible, the narrative/qualitative synthesis will be undertaken. As far as possible, we will rely on data reported in the individual SR. For rare events, we anticipate that it may be necessary to reanalyze the data so comparable data are presented in the overview.

### 2.6 Subgroup analysis

If the necessary data are available, subgroup analyses will be conducted for specific cancer types (lung cancer, breast cancer, prostate cancer, and others), and other study or patients characteristics.

### 2.7 Quality assessment

We plan to assess the reporting and methodological quality of the included SRs/MAs. Two review authors to assess quality independently. Discrepancies can be resolved through discussion.

#### 2.7.1 Reporting quality assessment

The Preferred Reporting Items for Systematic Reviews and Meta-analyses (PRISMA) checklist including 27 items in 7 parts.^[22]^ In the PRISMA, each item has 3 answer options: yes, no, and partial yes. We will use PRISMA checklist to assess each item of the included SRs/MAs. Each of the items will be scored “1” for yes, “0.5” for partial yes, and “0” for no. We will judge the reporting quality based on the score of each SRs.

#### 2.7.2 Methodological quality assessment

We will use the “A Measurement Tool to Assess Systematic Reviews" (AMSTAR) 2 instrument to assess the methodological quality of included SRs/MAs. This updated version form the original AMSTAR tool and it allows for the appraisal of SRs/MAs of randomized and nonrandomized studies of interventions, which based on 16 items, 7 of which are critical (items: 2, 4, 7, 9, 11, 13, and 15) and 9 of which are noncritical.^[23]^ We will assess each review against the 16-item instrument. An overall rating of quality will be assigned according to the algorithm suggested by Shea et al.^[23]^ AMSTAR-2 classifies the quality of an SR as high, moderate, low, or critically low. A review with less than or equal to 1 noncritical weakness is classified as being of high quality; a review with more than 1 noncritical weakness will be classified as being of moderate quality. A review with one critical flaw or (and) with multiple noncritical flaws will be classified as being of low quality and one with >1 critical flaw is classified as being of critically low quality.

## 3 Discussion

Exercise or sport activities have been recommended as part of standard care for patients with cancer to help prevent and manage physical and psychosocial problems, and improve QoL. Our study will include all published SRs and MAs of exercise or sport activities for patients with cancer. The results of our review will provide a comprehensive description on reporting and methodological quality of existing SRs/MAs and effects of exercises or sport activities for cancers.

## Author contributions

Conceptualization: Fang-fang Wang, Yang Yuan, Min Wang. Investigation: Fang-fang Wang, Shui-lin Wu. Methodology: Fang-fang Wang, Yang Yuan, Xiu-xiu Deng. Resources: Yu-jun Song, Yan-qiong Wu, Yun He, Ding-mei Dai. Writing – original draft: Fang-fang Wang. Writing – review & editing: Yang Yuan, Min Wang.
